# Consensus classification of biliary complications after liver transplantation: guidelines from the BileducTx meeting

**DOI:** 10.1093/bjs/znae321

**Published:** 2025-05-02

**Authors:** Hannah Esser, Iris E M de Jong, Floris M Roos, Christina Bogensperger, Stefan M Brunner, Benno Cardini, Philipp Dutkowski, Hasan Eker, Sofia Ferreira-Gonzalez, Stuart J Forbes, Peter J Friend, Yiliam Fundora, Henrik Junger, Felix J Krendl, Paulo N Martins, Vincent E de Meijer, Rupert Oberhuber, Gabriel C Oniscu, Damiano Patrono, Robert J Porte, Thomas Resch, Hatem Sadik, Andrea Schlegel, Nicola De Stefano, Mathias Vidgren, Christopher J E Watson, Annemarie Weißenbacher, Stefan Schneeberger

**Affiliations:** Department of Visceral, Transplant and Thoracic Surgery, organLife Laboratory, Centre of Operative Medicine, Medical University of Innsbruck, Innsbruck, Austria; Department of Medicine, University of Pennsylvania, Philadelphia, Pennsylvania, USA; Center for Engineering MechanoBiology, University of Pennsylvania, Philadelphia, Pennsylvania, USA; Wellcome-MRC Cambridge Stem Cell Institute, University of Cambridge, Cambridge, UK; Department of Visceral, Transplant and Thoracic Surgery, organLife Laboratory, Centre of Operative Medicine, Medical University of Innsbruck, Innsbruck, Austria; Department of Surgery, University Medical Center Regensburg, Regensburg, Germany; Department of Visceral, Transplant and Thoracic Surgery, organLife Laboratory, Centre of Operative Medicine, Medical University of Innsbruck, Innsbruck, Austria; Department of Surgery, Clarunis—University Centre for Gastrointestinal and Hepatopancreatobiliary Diseases, Basel, Switzerland and Department of Visceral Surgery, University Hospital Basel, Switzerland; Department for General and HPB Surgery and Liver Transplantation, Ghent University Hospital, Ghent, Belgium; Centre for Inflammation Research (CIR), University of Edinburgh, The Queen’s Medical Research Institute, Edinburgh, UK; Centre for Regenerative Medicine, University of Edinburgh, Edinburgh, UK; Nuffield Department of Surgical Sciences, University of Oxford, The Churchill Hospital, Oxford, UK; Department of Surgery. HPB and Liver Transplant Unit, ICMDM, Hospital Clinic Barcelona, IDIBAPS, UB, Barcelona, Spain; Department of Surgery, University Medical Center Regensburg, Regensburg, Germany; Department of Visceral, Transplant and Thoracic Surgery, organLife Laboratory, Centre of Operative Medicine, Medical University of Innsbruck, Innsbruck, Austria; Department of Surgery, Oklahoma University, Oklahoma City, USA; Department of Surgery, Section of Hepatobiliary Surgery and Liver Transplantation, University of Groningen, University Medical Center Groningen, Groningen, The Netherlands and UMCG Comprehensive Transplant Center, Groningen, The Netherlands; Department of Visceral, Transplant and Thoracic Surgery, organLife Laboratory, Centre of Operative Medicine, Medical University of Innsbruck, Innsbruck, Austria; Division of Transplantation Surgery, CLINTEC, Karolinska Institutet, Stockholm, Sweden; General Surgery 2U—Liver Transplant Centre, A.O.U. Città della Salute e della Scienza— Torino, Italy; Department of Surgery, Division of Hepato-Pancreato-Biliary and Transplant Surgery, Erasmus MC Transplant Institute, University Medical Center Rotterdam, Rotterdam, The Netherlands; Department of Visceral, Transplant and Thoracic Surgery, organLife Laboratory, Centre of Operative Medicine, Medical University of Innsbruck, Innsbruck, Austria; Nuffield Department of Surgical Sciences, University of Oxford, The Churchill Hospital, Oxford, UK; Transplantation Center and Department of Immunology, Lerner Research Institute, Cleveland Clinic, Cleveland, Ohio, USA; General Surgery 2U—Liver Transplant Centre, A.O.U. Città della Salute e della Scienza— Torino, Italy; Division of Transplantation Surgery, CLINTEC, Karolinska Institutet, Stockholm, Sweden; The Roy Calne Transplant Unit and the University of Cambridge Department of Surgery, Addenbrooke's Hospital, Cambridge, UK; Department of Visceral, Transplant and Thoracic Surgery, organLife Laboratory, Centre of Operative Medicine, Medical University of Innsbruck, Innsbruck, Austria; Department of Visceral, Transplant and Thoracic Surgery, organLife Laboratory, Centre of Operative Medicine, Medical University of Innsbruck, Innsbruck, Austria

## Introduction

Orthotopic liver transplantation (OLT) represents the standard of care for patients with end-stage liver disease, acute liver failure, and certain types of liver-related malignancies such as hepatocellular carcinoma^1^. Although in the initial phase following OLT patient survival is mainly determined by hepatocyte function and vascular complications^2–4^, long-term graft survival is often determined by biliary complications. Biliary complications are post-OLT complications affecting the biliary tract and occur in 20–40% of liver transplant recipients depending on the definition, reporting accuracy, experience, graft type, etc.^5–10^.

The pathogenesis of biliary complications is only partially understood, and the clinical implications can be severe; biliary complications often require multiple therapeutic interventions and can result in re-transplantation or even death^5,10^. Besides an increase in patient morbidity and mortality rates, biliary complications also translate into increased medical care costs^11,12^ and are the main cause for donation after circulatory death (DCD) liver transplantation to be 30% more expensive than donation after brain death (DBD) liver transplantation^13^.

The term biliary complications encompasses a plethora of complications affecting the biliary tract including biliary leakage, anastomotic strictures, and non-anastomotic strictures (NAS). NAS are regarded as one of the most troublesome biliary complications as they often remain therapy-resistant and frequently result in graft loss^5,14^. NAS are diagnosed in up to 44% of recipients of DCD liver grafts and in about 5% of recipients of DBD liver grafts^8,15^.

Consistency in how to diagnose and report biliary complications in OLT is currently lacking and therefore clinical studies are often non-comparable, as reflected by the high variability in NAS incidence across different studies, hampering advances in the field. We here report the results of the consensus voting and discussing as part of the BileducTx meeting held in Innsbruck on 14–15 December 2023. These guidelines provide clarity on the definition, grading, monitoring, and reporting of post-OLT biliary complications aiming to facilitate future clinical trial development.

## Methods

A faculty was chosen based on their expertise and publication record in the field of liver transplantation and biliary (patho)physiology from both clinical as well as more fundamental backgrounds. Following a formal review of the topic at the conference, the experts were asked to vote on statements regarding the definition and monitoring of post-OLT biliary complications followed by an open discussion. After the conference, refined statements were then sent to the experts for online voting according to a modified Delphi methodology (agree/disagree, make recommendations for changes). Statements were agreed on or dismissed based on an 80% consensus threshold. Three rounds of online voting were undertaken followed by an additional round of online discussion^16^.

## Overview of biliary complications after liver transplantation and current definitions

Biliary complications encompass any complication after OLT involving the biliary tract. These range from biliary leaks at the anastomosis to strictures at any other location, either with or without intrahepatic biloma, prestenotic dilations, vanishing ducts, recurrent cholangitis, or biliary casts and/or sludge^17,18^. This heterogeneity in presentation and location of biliary complications as well as the different post-OLT time intervals used for biliary complications assessment have led to inconsistencies in the literature.

In addition, radiological appearance and clinical pictures can be similar for biliary complications of different aetiologies, further complicating proper classification. For example, biliary strictures due to recurrence of primary sclerosing cholangitis, hepatic artery thrombosis, or post-ischaemic and immune-mediated injuries are generally indistinguishable on cholangiography (either endoscopic retrograde cholangiopancreatography (ERCP) or magnetic resonance cholangiopancreatography (MRCP)).

Similarly, the clinical presentation of NAS can be mimicked by other processes: anastomotic strictures that, if ignored or insufficiently treated, may progress to (diffuse) non-anastomotic strictures; or cholangitis after OLT may develop from already existing cholestasis following increased bile viscosity damaging the bile duct wall and contributing to the development of NAS.

Therefore, it may be challenging to pinpoint the exact aetiology in individual patients, rendering terms such as ischaemic-type biliary lesions or ischaemic cholangiopathy impractical. To overcome this problem, it has been proposed to use the more general term ‘post-transplant cholangiopathy’. We support the use of this terminology and will use post-transplant cholangiopathy to indicate strictures or other complications that develop at any location in the biliary tree other than the anastomosis with an intact vascular supply.

**Keypoints**

- Consistency in how to diagnose and report biliary complications in OLT is currently lacking.
- A consensus on how to define and report biliary complications after OLT is urgently needed to guide future clinical trial designs and improve post-OLT outcomes.

## The pathogenesis of biliary complications

### Ischaemia-mediated injury leading to biliary complications

During an OLT procedure, the donor liver and bile ducts are subjected to unphysiological conditions; following confirmed death of the donor and subsequent organ procurement, the liver is usually transferred to the recipient hospital on ice or using *ex situ* machine perfusion devices. During procurement and transport, the liver and bile ducts undergo a period of warm and cold ischaemia in case of DCD or cold ischaemia alone for DBD livers. These periods of ischaemia are followed by reperfusion upon completion of the vascular anastomoses in the recipient^19^. This re-oxygenation during reperfusion after ischaemia causes an influx of cytokines and inflammatory cells in the liver and bile ducts, initiating an extensive wound-healing response^20^. Within the cells, mitochondria are key effectors of ischaemia–reperfusion injury (IRI). The tricarboxylic acid cycle and electron transport chain arrest during ischaemia, resulting in depletion of ATP and accumulation of succinate and NADH^21^. Upon reperfusion and, thus, re-introduction of oxygen, the excess of succinate is oxidized at an increased rate stimulating reactive oxygen species (ROS) production by undirected electron transport in mitochondrial complex I^21–24^. Relative low levels of ROS can be scavenged by antioxidants, maintaining the redox balance, but severe oxidative stress drives the cell into apoptosis or even necrosis^25^. In case of the latter, damage-associated molecular patterns are released, recruiting immune cells and initiating an inflammatory response^26–28^. IRI is especially detrimental for cholangiocytes as less antioxidants to scavenge ROS are available in (large) cholangiocytes compared to hepatocytes^29–31^. This may explain the extensive damage to the biliary surface epithelium after static cold storage; over 90% of the donor livers lose the majority of surface epithelium in the distal extrahepatic bile duct^32–35^.

Longer warm and cold ischaemia times are associated with more severe histological bile duct damage, translating into a higher risk of developing post-transplant cholangiopathy^32–34,36,37^. This means that the extent of biliary damage during ischaemia—the (still) unavoidable part of OLT—plays a role in the development of biliary complications and especially post-transplant cholangiopathy. It is critical that the bile ducts can regenerate and restore function after OLT; however, if severe biliary damage after IRI results in unsuccessful regeneration and ongoing inflammation, biliary strictures may develop in the recipient.

Epithelial regeneration following damage is achieved by the remaining cholangiocytes lining the luminal surface (that is surface epithelium) and those within the submucosa of the large bile ducts. The cholangiocytes within the submucosa are organized in acini clusters^38,39^, which are called peribiliary glands (PBGs). Cholangiocytes are a highly heterogeneous cell population that display distinct characteristics depending on their localization within the biliary system^40–44^. Small ductules of the intrahepatic bile ducts are lined by 4–5 cuboidal cholangiocytes per circumference. With the consecutive enlargement of the bile ducts, cholangiocytes become larger in size and more columnar^45–47^. Following damage of large cholangiocytes, small cholangiocytes can acquire a large cholangiocyte phenotype and replenish them^48,49^.

The molecular pathways driving repair of the biliary tree following IRI are very complex and multiple mechanisms may hamper or prevent adequate restoration of the ducts, favouring fibrosis as opposed to regeneration. One important factor influencing biliary regeneration is cellular senescence^50,51^, which is defined as irreversible cell cycle arrest accompanied by a characteristic change in phenotype^52^. Recently, it was shown that biliary cellular senescence compromised adequate biliary regeneration in the setting of OLT. Cellular senescence was triggered in cholangiocytes during experimental liver cold storage, which negatively affected cholangiocyte proliferation. Administration of a senolytic drug prior to cold storage preserved biliary architecture and improved biliary regeneration^36,51^.

Cholangiocyte regeneration and function require a sufficient oxygen supply^53^. The cholangiocytes’ vascular supply depends on the integrity of the peribiliary plexus arising from the hepatic artery^54^. IRI can cause damage to the peribiliary vascular plexus resulting in subintimal oedema or arteriolo-necrosis^33^. Furthermore, ischaemia can lead to fibrin deposition in the peribiliary vascular plexus^55–57^ and thus impair regeneration. Using D-dimer flush out during *ex situ* normothermic machine perfusion as a surrogate for fibrin depositions, it has been shown that D-dimer levels correlate with the duration of cold ischaemia in DBD liver grafts and also with poor transplant outcomes^56,57^. High D-dimer levels are also associated with the development of biliary complications^57^. The importance of microvascular fibrin deposits in biliary complications development is further supported by the observation, that livers being subjected to fibrinolytic treatment have lower post-transplant cholangiopathy rates, suggesting fibrinolysis as a new strategy to improve post-OLT outcomes^57^.

As PBGs are often the only cholangiocyte compartment in the (distal) extrahepatic bile ducts that survive severe IRI (thus tasked with regenerating the lost cholangiocytes^58^), the PBG niche has been studied in more detail to understand the pathophysiology of biliary complications. PBGs differ from the cholangiocytes in the surface epithelium in at least location, morphology, and metabolism. The deeper location in the submucosa protects PBGs from the harsh luminal environment including toxic bile salts. In addition, their glycolytic metabolism, in contrast to an oxidative metabolism, renders them relatively resistant to hypoxia^53^. PBGs produce vascular endothelial growth factor upon ischaemia that promotes PBG as well as endothelial cell expansion^59^. Extensive damage to the endothelium or peribiliary vascular occlusion by fibrin thrombi interferes with this physiological mechanism, leading to ongoing local hypoxia which prevents adequate epithelial regeneration^53^. Of note, during this period of biliary wound healing, serum markers for biliary obstruction such as gamma glutaryl transferase (GT), alkaline phosphatase and direct bilirubin can fluctuate indicating active regeneration^60^.

If restoration of the protective surface epithelium is delayed or impaired, toxic bile may enter the biliary submucosa, aggravating damage^61–63^. Under physiological circumstances, biliary epithelium modifies bile and promotes bile flow by the secretion of water and bicarbonate^64^. Bicarbonate secretion is extremely important to maintain an alkaline milieu apical of the cholangiocyte layer. Adult cholangiocytes carry a glycocalyx on their apical membrane that can trap bicarbonate molecules ^65^. Bicarbonate deprotonates toxic hydrophobic bile salts and thereby provides a chemical barrier to protect the biliary surface epithelium; this is called the ‘bicarbonate umbrella’^66^. One important electrolyte transporter involved in maintenance of the bicarbonate umbrella is the cystic fibrosis transmembrane conductance regulator (CFTR), which secretes chloride into the bile. Chloride is subsequently reabsorbed in exchange for bicarbonate by the anion exchange pump 2 (AE2). Hypoxia has been shown to decrease CFTR activity in cholangiocyte organoids, suggesting impairment of the bicarbonate umbrella following ischaemia, which could aggravate biliary injury^67^.

Another protective mechanism against toxic hydrophobic bile salts is the formation of mixed micelles consisting of both hydrophobic bile salts and phospholipids^68^. After OLT, the bile salt export pump (secretion of bile salts) and multidrug resistance 3 (MDR3, secretion of phospholipids) transporters both need time to recover, albeit MDR3 recovers at a slower pace resulting in a high bile salt-to-phospholipid (BS/PL) ratio directly after OLT^61–63^. A high BS/PL ratio results in increased levels of free hydrophobic bile salt monomers, exposing cholangiocytes (or bare submucosa at places where the adult surface epithelium is not yet recovered) to cytotoxic bile. The significance of this mechanism is demonstrated by the positive correlations between an increased BS/PL ratio, longer ischaemia times, severe histological damage, and the development of post-transplant cholangiopathy^61–63^.

**Fig. 1:**
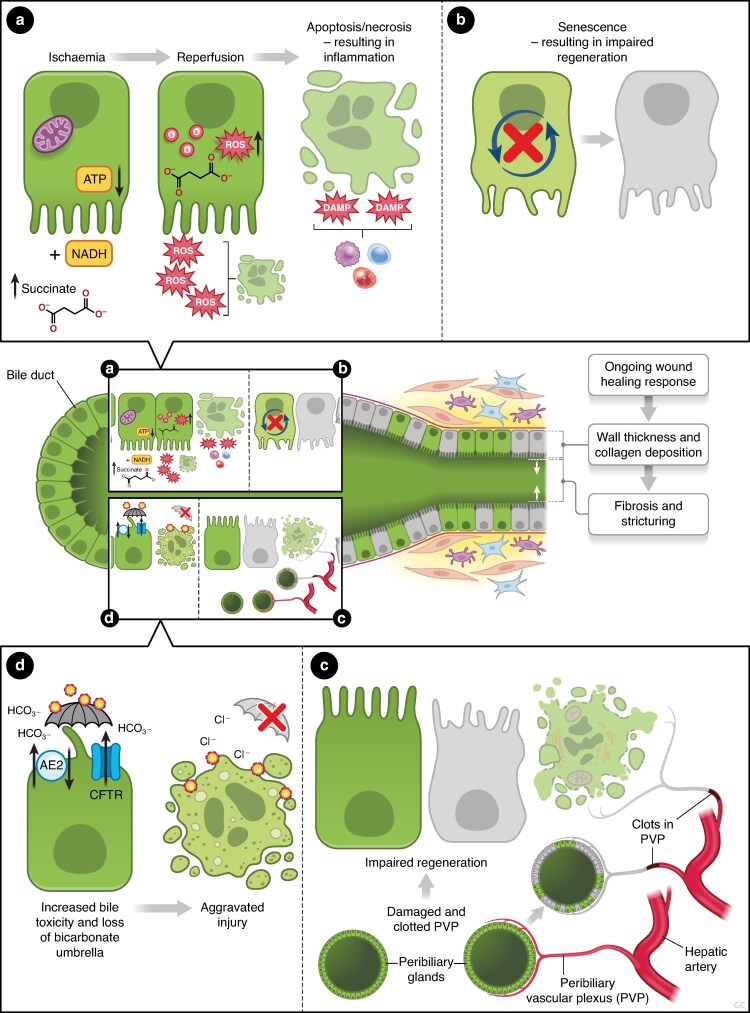
Factors contributing to the development of post-transplant cholangiopathy During the procurement and transplant process cholangiocytes are exposed to ischaemia-mediated injury resulting in damage to mitochondria and the subsequent release of reactive oxygen species (ROS). If ROS can’t be scavenged by antioxidants, cholangiocytes can become apoptotic or necrotic and contribute to inflammatory processes by releasing cytokines **a**. The majority of (distal) cholangiocytes suffers profound damage during the liver transplant process. Lost surface epithelium needs to be repaired by the surviving cholangiocyte population. The regenerative capacity of the remaining cholangiocytes is negatively impacted by factors such as cellular senescence, an irreversible cell cycle arrest preventing cholangiocyte proliferation and contributing to inflammation **b**, and local hypoxia caused by damage to the peribiliary vascular plexus **c**. If the restoration of the surface epithelium is impaired, increased bile toxicity and collapse of the bicarbonate umbrella aggravate biliary injury **d**, triggering an ongoing wound-healing response which results in fibrosis and stricturing.

The above mechanisms suggest that severe biliary damage may result in a cholangiocyte pool that is either too small to adequately regenerate and/or dysfunctional by senescence, ongoing local hypoxia, peribiliary vascular occlusion, or bile salt toxicity promoting scarring and strictures over epithelial regeneration (*Fig. 1*, *Table 1*).

**Table 1:** Mechanisms underlying the development of post-transplant cholangiopathy

**Ischaemia-mediated injury (typically early after OLT)**
Inadequate biliary regeneration after damage resulting from (a combination of): Decrease of the biliary regenerative capacity: ˚Extensive biliary damage leaving only a small number of viable cholangiocytes after OLT ˚Damage to the peribiliary glands, vascular plexus and supporting stroma ˚Severe cholangiocyte senescence Ongoing biliary injury: ˚Continued local hypoxia due to damage to or occlusion of the vascular plexus ˚Bile salt toxicity
**Immune-mediated injury typically later after OLT)**
ABO-incompatibility Immune-related hepatobiliary diseases such as auto-immune hepatitis and primary sclerosing cholangitis Donor CMV infection A mutation in chemokine receptor CCR5

CCR5, C-C chemokine receptor type 5; CMV, cytomegalovirus; OLT, orthotopic liver transplantation.

### Immune-mediated injury causing biliary complications

Post-transplant cholangiopathy that occurs more than one year after OLT has been associated with immune-mediated injuries rather than ischaemia^69^. It often involves the smaller bile duct branches in the periphery as opposed to the larger ducts. Multiple immune-related variables were correlated with this late-type post-transplant cholangiopathy; ABO-incompatibility^70,71^, immune-related hepatobiliary diseases such as autoimmune hepatitis and primary sclerosing cholangitis, donor cytomegalovirus infection, and a mutation in chemokine receptor CCR5^72,73^ (*Table 1*).

**Keypoints**

- Low levels of antioxidants render cholangiocytes especially vulnerable to ischaemia–reperfusion injury.
- The majority of biliary epithelium suffers profound damage during the transplant process.
- It is critical that the bile ducts can regenerate and restore function following transplantation into the recipient.
- Ischaemia induces fibrin deposition in the peribiliary vascular plexus causing local stromal infarcts, damaging epithelium and peribiliary glands
- Multiple mechanisms may prevent adequate restoration of the ducts favouring fibrosis as opposed to regeneration.

### Surgical injury leading to biliary complications

About 25% of liver transplant recipients develop anastomotic strictures^5,74,75^. These strictures are thought to result from factors related to the procedure^76^. Anastomotic strictures can be divided into early (within 6 months post-OLT) and late anastomotic strictures (after 6 months post-OLT)^77^. Almost 70% of anastomotic strictures present as early anastomotic strictures, emphasizing a surgery-related cause (for example poor tissue perfusion, suturing technique). Some anastomotic strictures occur at a later point in time, and these may result from local ischaemia at the site of the anastomosis preventing adequate regeneration and the formation of a fibrotic scar instead. It is therefore critical to avoid skeletonization of the ducts so that they are surrounded by sufficient tissue during the transplant procedure to preserve the vascular network. Other factors that may contribute to the formation of an anastomotic stricture are a size mismatch of the donor and recipient duct, hepatic artery thrombosis, sex mismatch, anastomotic bile leakage, or a liver transplantation using a split graft or a liver from a living donor^74,76^. All these factors cause either local ischaemia around the anastomosis, increased biliary damage, or size discrepancy between the ducts, leading to inadequate regeneration and fibrotic scar formation.

## Reporting guidelines

After the in-person meeting, three rounds of online voting, and a subsequent online discussion, the panel agreed on the following recommendations for classifying and reporting biliary complications.

### What classification should be used to report biliary complications?

**Fig. 2:**
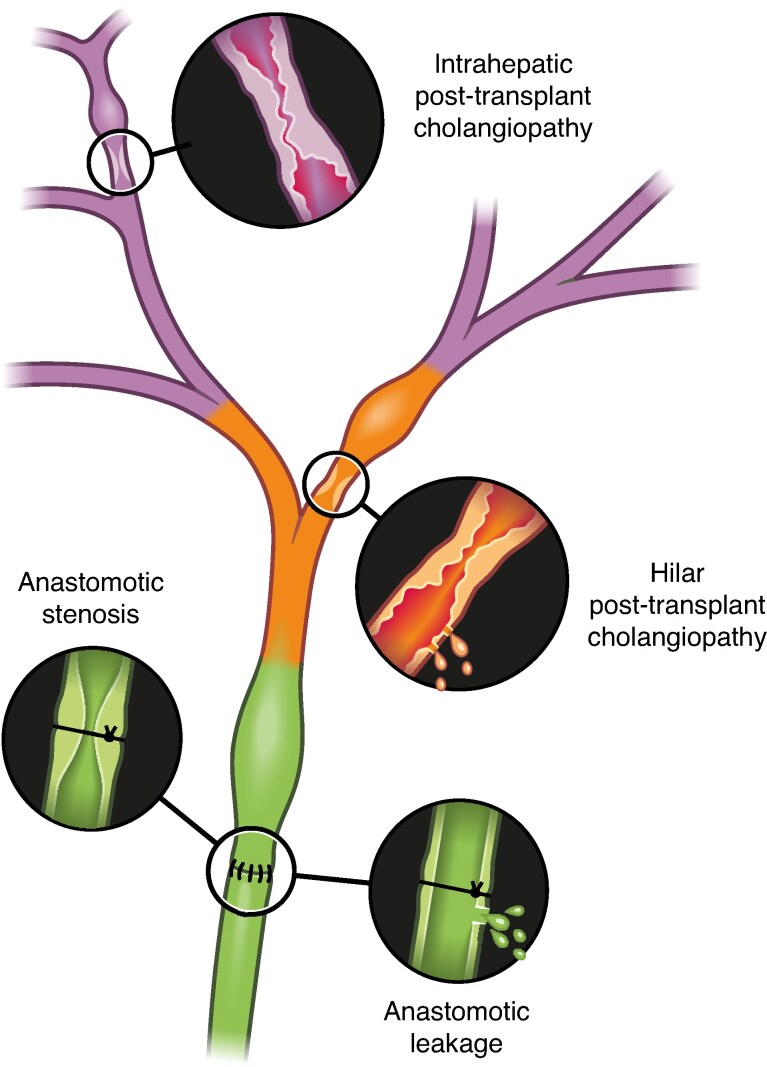
Classification of biliary complications following liver transplantation

As described above, it can be challenging to distinguish biliary complications of different aetiologies. Thus, the panel suggests that the term *post-transplant cholangiopathy* should be used for all biliary complications not affecting the anastomotic region. Post-transplant cholangiopathies should then further be divided into *intrahepatic and hilar* post-transplant cholangiopathy (*Fig. 2*). The hilar region is defined as comprising the first-order branch of the biliary tree up to (but not including) second-order branches.

Biliary complications affecting the anastomotic region should be reported as *anastomotic stricture or leakage*.

All other post-OLT complications related to the biliary tree such as cystic stump insufficiencies and aberrant bile ducts should be reported separately and not included in the overall biliary complications rate.

When reporting biliary complications rates the type of biliary reconstruction (duct-to-duct anastomosis, biliodigestive anastomosis, etc.) should be specified. The occurrence of arterial complications should likewise be reported.

**Recommendations**

- Biliary complications should be categorized according to their *localization in the biliary tree*. The three categories established as part of this consensus meeting are:
  1. *Intrahepatic post-transplant cholangiopathy*; no requirement to differentiate between stricture/leakage
  2. *Hilar post-transplant cholangiopathy*; no requirement to differentiate between stricture/leakage
  3. *Anastomotic complications*; anastomotic complications should be reported as anastomotic strictures or leakage
- Biliary complications such as cystic stump insufficiencies or aberrant bile ducts should be reported separately and not be included into the general biliary complications rate.
- The type of biliary reconstruction should be reported.
- Arterial complications and primary sclerosing cholangitis/ autoimmune hepatitis as an indication for liver transplantation should be reported.
- The timing of occurrence of biliary complications should be reported, as well as the follow-up time of OLT recipients.

### How should biliary complications be reported?

Similarly to general surgical complications^78^, post-OLT biliary complications should be graded according to their clinical severity.

**Fig. 3:**
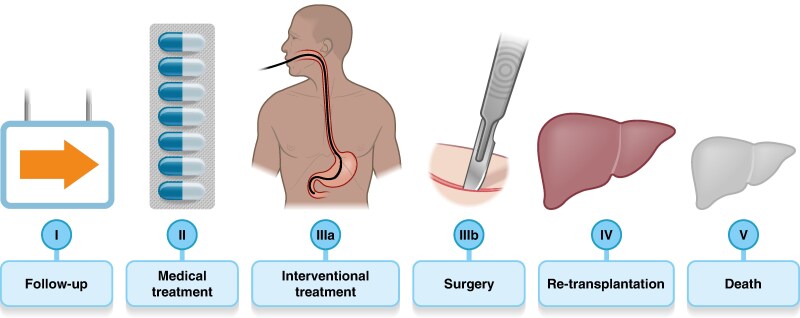
Suggested classification for grading of biliary complications following liver transplantation

The panel established a modified Clavien–Dindo classification^78^ for post-OLT biliary complications that could aid in consistent reporting and facilitate future clinical trial design (*Fig. 3*).

**Recommendations**

Biliary complications should be reported with their *location* (intrahepatic post-transplant cholangiopathy, hilar post-transplant cholangiopathy, anastomotic stricture/leakage) and *graded* according to a newly established classification.

- Radiological abnormalities on imaging (‘watch and wait’ policy)*
- Biliary complications requiring medical treatment (for example antibiotics, ursodeoxycholic acid, etc.)
- Biliary complications requiring
  - Endoscopic or radiologic intervention (for example endoscopy and stent, percutaneous transhepatic biliary drainage (PTBD))
  - Surgical intervention (other than re-transplantation)
- Biliary complications requiring re-transplantation
- Patient death due to biliary complications

* Indication for imaging should be reported, for example ‘abnormalities on protocol imaging’ or ‘increase in cholestatic liver function tests for which additional imaging was indicated’.

### What timepoint should be used for assessment of the biliary complication rate?

To facilitate clinical trial design the panel recommends assessment of the biliary complications rate at 12 months post-OLT. This is based on the observation that by 12 months post-OLT the majority of IRI-related cholangiopathies has manifested, while longer follow-up intervals increase the chance of misclassifying immune-related complications such as recurrent primary sclerosing cholangitis (PSC) as biliary complications.

The highest-grade biliary complication occurring during the first 12 months post-OLT should be reported. In addition, in case of longer follow-up the highest-grade biliary complication occurring throughout the median follow-up time (exceeding 12 months) should be reported.

**Recommendations**

- The rate of biliary complications should be assessed 12 months post liver transplant
- The highest-grade biliary complication, that occurred within the first 12 months post liver transplant should be reported.

### Which imaging modality should be used?

To reach consensus on the preferred imaging modality for the diagnosis of biliary complications several rounds of discussions were required. Finally, the consensus was reached, that MRCP/ERCP represent the gold standard for diagnosis of biliary complications. As standard procedures and resources vary widely between hospital (for example presence/absence of protocol imaging at different time points; availability of imaging modalities; high costs) it was agreed that no protocol MRCP/ERCP is required to confirm the absence of biliary complications following OLT.

**Recommendations**

- The gold standard for diagnosis of biliary complications is MRCP/ERCP.
- However, as resources and standard operating procedures vary between hospitals no protocol MRCP/ERCP at a set timepoint is required to confirm the absence of biliary complications following liver transplant.

### Should arterial complications and recurrence of PSC be excluded prior to diagnosis of a biliary complication?

Arterial complications can lead to the development of biliary strictures and should therefore be excluded prior to assessing biliary complication rates. Any arterial complication should be described when reporting biliary complication rates.

Similarly, portal vein complications (particularly low portal venous flow and intraoperative hypotension) can contribute to biliary complications development and should therefore be reported.

Recurrent PSC and chronic rejection should be excluded prior to the diagnosis of biliary complications. As it can be difficult to differentiate between recurrent PSC and cholangiopathy, the panel recommends reporting the presence of PSC or other immune-related diseases in the recipient population.

**Recommendations**

- Arterial complications should be excluded prior to the diagnosis of biliary complications. Likewise, recurrence of PSC or chronic rejection should be excluded.*

*As it can be difficult to differentiate between chronic rejection, recurrent PSC and post liver transplant cholangiopathy, the presence of PSC and other immune-related diseases in the recipient population should be reported.

## Data Availability

Not applicable.
